# Efficacy of BRS^®^ and Alpron^®^/Bilpron^®^ Disinfectants for Dental Unit Waterlines: A Six-Year Study

**DOI:** 10.3390/ijerph17082634

**Published:** 2020-04-12

**Authors:** Alexandre Baudet, Julie Lizon, Jean-Marc Martrette, Frédéric Camelot, Arnaud Florentin, Céline Clément

**Affiliations:** 1 Faculty of Dentistry, University of Lorraine, 54505 Vandœuvre-lès-Nancy, France; jean-marc.martrette@univ-lorraine.fr (J.-M.M.); celine.clement@univ-lorraine.fr (C.C.); 2 Department of Dentistry, University Hospital, 54000 Nancy, France; 3 Department of Hygiene and Environmental Analysis, University Hospital, 54505 Vandœuvre-lès-Nancy, France; j.lizon@chru-nancy.fr (J.L.); arnaud.florentin@univ-lorraine.fr (A.F.); 4 EA 3450 DevAH, University of Lorraine, 54505 Vandœuvre-lès-Nancy, France; 5 Dental Private Practice, 88300 Neufchateau, France; frederic.camelot@wanadoo.fr; 6 Faculty of Medicine, University of Lorraine, 54505 Vandœuvre-lès-Nancy, France; 7 EA 4360 APEMAC, University of Lorraine, 54505 Vandœuvre-lès-Nancy, France

**Keywords:** water quality, infectious control, dental chair, waterlines, water microbiology

## Abstract

Biofilms in dental unit waterlines (DUWL) are a potentially significant source of contamination posing a significant health risk as these may come into contact with patients and dental staff during treatment. The aim of this study was to evaluate the microbiological quality of DUWL water treated by Biofilm-Removing-System^®^ (BRS^®^) and Alpron^®^/Bilpron^®^ disinfectant solutions for six years in a French university hospital. The microbiological quality of water supplied by 68 dental units—initially shock treated with BRS^®^, then continuously treated by Alpron^®^ with sterile water during working days and Bilpron^®^ during inactivity period, and combined with purging every morning and after each patient—was assessed biannually during six years for total culturable aerobic bacteria at 22 °C and 36 °C, *Legionella* sp., *Pseudomonas aeruginosa*, and total coliforms. A total of 628 samples were analyzed, 99.8% were compliant with extended microbiological levels, and we never detected pathogen bacteria like *Legionella* sp. and *P. aeruginosa*. Only one sample (0.2%) was noncompliant with the level of total culturable aerobic bacteria at 36 °C, which exceeded 140 colony forming units per mL. The protocol implemented in our university hospital gives excellent results and enables control of the microbiological quality of DUWL water in the long term.

## 1. Introduction

Water is essential in dental units for rotary equipment and mouth rinse. Nevertheless, the microbiological quality of dental units’ water is often poor compared to that of drinking water sources [1,2,3]. This water quality is important since patients and dental staff are regularly exposed to water and aerosols generated during dental treatments. Dental unit waterlines (DUWL) contaminated with *Legionella pneumophila* have caused the death of an Italian [4] and a Swedish patient [5]. Two immunocompromised patients have presented a dental abscess associated with *Pseudomonas aeruginosa* after exposure to DUWL water contaminated by this microorganism [6]. Groups of children developed infections with *Mycobacterium abscessus* after having pulpotomy in a hospital in which DUWL were contaminated by this nontuberculous mycobacterium [7,8]. Infectious risk can also affect the healthcare workers [9,10,11].

Microbiological water contamination of DUWL may be due to the water supply [12,13,14], to the retrograde contamination by oral fluids [13,15,16], and to the formation of a biofilm within the tubing promoted by the strong complexity of DUWL, the low water flow in DUWL with a negligible flow at the periphery of the lumen [14,17], the plastic materials constituting the DUWL [18], and the water stagnation during inactivity periods [14,19]. In all cases, the infectious hazard can be limited by the implementation of a preventive strategy.

In the last 10 years, different DUWL disinfectants were tested in experimental conditions [20,21,22,23,24,25] or under real conditions of dental care during a few weeks [26,27] or months [28,29,30,31,32,33] but seldom after long-term application [34]. Up to now, the effectiveness of Alpron^®^ disinfectant was only evaluated during a few weeks of use in laboratory models [35] and in real working conditions, continuously [31,36,37] and intermittently [38], but never in the long term or with a large number of dental units.

In 2012, we began to analyze the outlet water of DUWL in the dental department of the regional university hospital of Nancy, France. The first analyses highlighted high microbiological water contamination consisting of aerobic mesophilic flora at 22 °C and 36 °C (≥300 colony forming units per mL (CFU/mL)) and *Legionella* sp. In response, different corrective actions have been implemented in order to standardize the water quality delivered by the DUWL [31].

The aim of this study was to evaluate the microbiological quality of DUWL water treated by Biofilm-Removing-System^®^ (BRS^®^) and Alpron^®^/Bilpron^®^ (ALPRO^®^ MEDICAL GmbH, Germany) disinfectant solutions for six years (from June 2013–June 2019) in the regional university hospital of Nancy, France.

## 2. Materials and Methods

### 2.1. Dental Units

The dental department of the university hospital of Nancy owns different sites which changed during the following period: site A with 44 dental units, site B with six dental units closed in 2016 and replaced by site C with 16 new dental units, and site D with two and then one dental unit since 2017. The dental chairs were manufactured by A-Dec^®^ (A-Dec Inc., Newberg, OR, USA) and date from 2005, except for the 16 chairs of site C which date from 2016. All our DUWL were disconnected from the hospital main water supply and an independent tank—a bottle—was added to each. The water supply bottles were disinfected daily in a thermal washer-disinfector. All DUWL were flushed for 30–45 s every morning and for 20–30 s after each patient. Our DUWL were progressively treated in the following way since 2013.

### 2.2. BRS^®^ and Alpron^®^/Bilpron^®^ Disinfectants

Initially, we removed the biofilms of the DUWL with BRS^®^ (ALPRO^®^ MEDICAL GmbH, Germany) according to NF EN ISO 16954. BRS^®^ consists of a 2-phase basic cleaning system to be put in the DUWL in this order: BRS^®^ PreCleaner (an enzymatic cleaning agent), then BRS^®^ Remover mixed with BRS^®^ Activator, followed by rinsing with sterile water and disinfection with Bilpron^®^ (ALPRO^®^ MEDICAL GmbH, Germany) for 12 h contact time. BRS^®^ was used once in all our DUWL at the beginning of the study, and it was reused in the contaminated DUWL after a noncompliant microbial control. Then, the water of DUWL was continuously treated with Alpron^®^ (ALPRO^®^ MEDICAL GmbH, Germany) and Bilpron^®^ disinfectant solutions during activity and inactivity periods (>24 h), respectively. Alpron^®^ is mainly made up of ethylene diamine tetra acetic acid (EDTA), polyaminopropyl biguanide, and sodium tosylchloramide. As water treatment, Alpron^®^ was used daily at 1% concentration in the water supply bottle with sterile water. The dilution of Alpron^®^ was performed in the one-liter supply bottles—with 10 mL of Alpron^®^ concentrate and 990 mL of sterile water—by dental staff (dental assistants, dental students, and dentists) after a hand disinfection. Bilpron^®^ is a disinfectant used unmixed during inactivity periods to inhibit the development of the biofilm in DUWL. In agreement with the manufacturer, it was used pure in bottles and DUWL for any period of inactivity exceeding 24 h. During the weekends and the holidays, Bilpron^®^ was used in all the DUWL. Bilpron^®^ contains EDTA, ester p-hydroxybenzoate, and polyhexamethylene biguanide.

### 2.3. Sampling

Water samples (500 mL) were taken simultaneously from the water syringe and from all instruments without pre-flushing. They were collected biannually during working hours—in the middle of the day—by a specialized team. Water samples were collected aseptically in sterile containers with a filtered mixture (20 mL) of sodium thiosulfate, Tween^®^ 80 (Sigma-Aldrich Inc., St Louis, MO, USA), lecithin, and histidine to inhibit the Alpron^®^ disinfectant. Samples were transferred to the laboratory within 2 h in insulated boxes and quickly processed in the following way.

### 2.4. Microbiological Control

Each water sample was subjected to analysis for total culturable aerobic bacteria, *Legionella* sp., *Pseudomonas aeruginosa*, and total coliform bacteria including *Escherichia coli* in the Laboratory of Environmental Biology of the university hospital.

Total culturable aerobic bacteria counts were performed according to the international standard for water quality: enumeration of culturable microorganisms—colony count by inoculation in a nutrient agar culture medium (NF EN ISO 6222 [39]). In brief, two samples of 1 mL of water were placed in two sterile 90 mm plastic petri dishes, followed by the addition of 20 mL of plate count agar (PCA) to each plate, and mixed well. The agar was allowed to harden at room temperature. Thus, one plate was incubated at 36 ± 2 °C and the other was incubated at 22 ± 2 °C for 44 ± 4 and 68 ± 4 h respectively. The colonies on each plate were counted immediately after incubation.

*Legionella* sp. and *L. pneumophila* detection was carried out according to the international standards for water quality: enumeration of *Legionella* (NF EN ISO 11731-2 [40]). Briefly, 0.2 mL of water was inoculated directly to glycine–vancomycin–polymyxin–cyclohexamide (GVPC) agar. Then, 100 mL and 10 mL of water were separately filtered through a black membrane made of mixed esters of cellulose (pore size 0.45 μm). The filters were overlaid with 30 mL of pH 2 acid for 5 ± 0.5 min. The filters were rinsed with 30 mL of sterile water and placed on GVPC agar plates. The cultures were incubated at 36 ± 2 °C for at least seven days, and CFU were counted at day three, day five, and day seven or more. Suspect colonies were subcultured on buffered-charcoal-yeast-extract (BCYE) agars with and without cysteine for one day.

Detection of *P. aeruginosa* was performed according to the international standard for water quality: detection and enumeration of *Pseudomonas aeruginosa*—method by membrane filtration (NF EN ISO 16266 [41]). In brief, 100 mL of water was filtered through a white membrane made of mixed esters of cellulose (pore size 0.45 μm), which was subsequently placed on Cetrimid agar (CN-agar) and incubated at 36 ± 2 °C for 44 ± 4 h. Colonies were counted and examined under UV radiation. The suspect colonies were tested with oxidase reagent and subcultured on King’s B medium.

Total coliform bacteria and *E. coli* were researched according to international standard for water quality: detection and enumeration of *Escherichia coli* and coliform bacteria—method by membrane filtration (NF EN ISO 9308-1 [42]). Briefly, 100 mL of water was filtered through a white membrane made of mixed esters of cellulose (pore size 0.45 μm), which was subsequently placed on a Lactose Triphenyl Tetrazolium Chloride (Lactose TTC) agar plate and incubated at 36 ± 2 °C for 44 ± 4 h. Colonies were counted, and suspect colonies were tested with oxidase reagent and subcultured in tryptophan broth tube for indole test.

The microbiological water quality levels were determined in our previous publication [31] according to the French guidelines about water in healthcare facilities [43]. These levels are presented in Table 1.

### 2.5. Statistical Analysis

Microbiological data were collected in Microsoft^®^ Excel (Microsoft Corporation, Redmond, WA, USA), and descriptive analyses were performed with RStudio^®^ (RStudio Inc., Boston, MA, USA) version 1.1.456.

## 3. Results

A total of 628 water samples were collected from 68 dental units between June 2013 and June 2019. During these six years, *Legionella* sp., *P. aeruginosa*, and total coliforms were never detected (Table 2). Two analyses of *Legionella* sp. were uninterpretable due to interfering flora, and 14 analyses of total coliforms were not realized; no other missing value is to be deplored. Concerning total culturable aerobic bacteria at 22 °C, all analyses were compliant with the limit of 300 CFU/mL: no colonies were retrieved in 573 samples (91.2%), 27 samples (4.3%) contained 1 CFU/mL, 27 samples (4.3%) contained between 2 and 100 CFU/mL, and one sample (0.2%) contained 156 CFU/mL (alert level). Concerning total culturable aerobic bacteria at 36 °C, one sample was noncompliant with the limit of 100 CFU/mL: no colonies were retrieved in 543 samples (86.5%), 38 samples (6.0%) contained 1 CFU/mL, 36 samples (5.7%) contained between 2 and 10 CFU/mL, 10 samples (1.6%) contained between 11 and 100 CFU/mL (alert level), and one sample (0.2%) contained 140 CFU/mL (action level).

Following the limits defined in Table 1, 617 samples (98.2%) were at target level, 10 (1.6%) were at alert level (total flora at 36 °C between 11 and 36 CFU/mL), and one (0.2%) was at action level (total flora at 36 °C: 140 CFU/mL while the total flora at 22 °C was 156 CFU/mL). Following the only noncompliant result in six years, the involved dental unit was temporarily taken out of service. These DUWL were flash treated with BRS^®^, and another analysis was performed to monitor the return to the target level before reuse.

## 4. Discussion

The continuous disinfection with Alpron^®^ 1% in sterile water during working days and unmixed Bilpron^®^ during periods of inactivity of at least 24 h has enabled to secure the water quality of our dental units. This procedure—complemented by a purge of the waterlines for 30–45 s at the beginning of the day and for 20–30 s after each patient and an initial treatment by BRS^®^—enables our university hospital to ensure patients’ safety. The water quality is crucial because patients can ingest and breath water during care. Furthermore, patients and dental staff are exposed to water by mucocutaneous contact due to projection and aerosols containing microorganisms [19,44]. Dental aerosols are able to disperse at more than one meter around the patient and to remain airborne for 20 min [45].

The good results of our study are subject to a collective and daily effort on the part of professionals. The dental staff refill several times a day the water supply bottles with the good dilution of Alpron^®^ in sterile water. They perform hand disinfection before manipulating the bottles. The water supply bottles are disinfected daily in a thermal washer-disinfector. The frequency of bottle disinfection is a key factor influencing the bacterial contamination of DUWL [13]. The morning purge enables to refresh the Alpron^®^ solution in the waterlines, and flushing after each patient helps to prevent patient fluids from going back up the DUWL and to avoid the cross-infection of successive patients [15,16]. Nevertheless, considering this long follow-up period, there cannot be any certainty as to the thoroughness of the compliance and the adherence to the procedures. This is especially because of the high turnover of dental students in our university hospital: almost 100 new students every year. Therefore, it is undeniable that flushing was not always performed, and it is possible that tap water was occasionally used instead of the sterile bottled water. Despite this, the obtained microbiological results are extremely satisfactory. However, these human errors are possibly the cause of the only noncompliant result and of the 10 samples at alert level obtained during the six years of follow-up. Indeed, operator errors and inappropriately applied protocols can account for the inconsistent results of dental unit disinfection [14,46,47].

This study was carried out in a university hospital divided into four operating spaces (sites A, B, C, and D), with both recent and old dental units used for different specific activities (restorative or prosthetic dentistry, endodontics, periodontics, dental surgery, pediatric odontology, and orthodontics). Despite this variability, the results highlight the perfect microbiological control of our DUWL.

The DUWL output water often contains multiple bacteria in quantities greater than what is recommended [3,44]. Among those bacteria, opportunistic pathogenic bacteria like *Pseudomonas* and *Legionella* can be found [19,48] despite DUWL disinfectants [30,34]. Before this protocol, the microbiological quality of DUWL’s water in our hospital was unsatisfactory and different punctual DUWL disinfectants were tested without prolonged success. Just before this new protocol, 50% of 44 DUWL sampled in our hospital was noncompliant with total culturable aerobic bacteria at 22 °C or 36 °C (>300 and >100 CFU/mL respectively). Also, one month before, seven DUWL were contaminated by *L. pneumophila* and one was contaminated by *P. aeruginosa* [31]. This is in accordance with the literature in which continuous disinfectants offer significantly better results than intermittent treatments [30,37]. The combination of a continuous treatment (Alpron^®^ 1%) used at low levels to minimize its potential toxic effect on patients associated with an intermittent treatment using a more concentrated active product (Bilpron^®^) has proven to be an effective method for the control of water contamination. The continuous treatment is effective in maintaining the total flora within the target level in DUWL output water [30], and the intermittent treatment enables to control the microorganisms that are otherwise resistant to disinfectant treatments because they are protected within the biofilm [49]. In addition, the intermittent treatment may be useful in preventing the adaptive resistance of bacteria that could be induced by continuous exposure to low concentrations of biocides [50]. The flash treatments are known to be ineffective in the long term, but they can quickly and significantly reduce bacterial contamination [29,30,31]; hence, we carried out an initial shock treatment with BRS^®^ to effectively decontaminate our DUWL just before implementing the continuous treatment.

A limitation of water disinfection is the use of chemicals, even though Alpron^®^ compounds seem innocuous for humans even if swallowed or for valve microtubes. We cannot guarantee the perfect safety of all chemical disinfectants on the exposed persons or on the environment. The presence of phenylalanine in Alpron^®^ forbids it for patients with phenylketonuria. Therefore, if physical treatment systems could permit a good microbiological quality of DUWL outlet water in the future, their use could be more environmentally friendly. In this way, for example, we can think of acoustic wave treatment; however, complementary studies are necessary [51].

Further study should evaluate the efficiency of Alpron^®^ with bacteriologically controlled water obtained by filtration of tap water. The use of filtered water with a 0.2-μm filter connected to a water faucet would avoid the handling, the storage, and the cost of the sterile bottled water.

## 5. Conclusions

In order to control the water contamination of DUWL, an internal control plan is necessary. The adopted control measures, including an initial shock treatment with BRS^®^, a combined continuous and intermittent disinfection with Alpron^®^/Bilpron^®^, sterile water, and regular flushing are effective in the long-term control of bacterial contamination. A surveillance of the microbiological quality of DUWL water is useful not only to assess the efficacy of preventive measures but also as a guide for the choice of corrective strategies.

## Figures and Tables

**Table 1:** Interpreting the results of dental unit microbial controls.

Results	Interpretation	Clinical Implication
Culturable aerobic flora at 36 °C ≤ 10 CFU/mL and Culturable aerobic flora at 22 °C ≤ 100 CFU/mL and Absence of pathogens (*L. pneumophila*, *P. aeruginosa*, etc.)	Target level Compliant	Continued use
Culturable aerobic flora at 36 °C > 10 and ≤ 100 CFU/mL or Culturable aerobic flora at 22 °C > 100 and ≤ 300 CFU/mL and Absence of pathogens (*L. pneumophila*, *P. aeruginosa*, etc.)	Alert level Compliant	Continued use with monitoring and/or preventive actions
Culturable aerobic flora at 36 °C > 100 CFU/mL or Culturable aerobic flora at 22 °C > 300 CFU/mL or Presence of pathogens (*L. pneumophila*, *P. aeruginosa*, etc.)	Action level Noncompliant	Prohibition of use, corrective actions, then new microbial analysis to control the return to the target level before reuse.

CFU—colony forming unit; °C—degrees Celsius; mL—milliliter; *L. pneumophila*—*Legionella pneumophila*; *P. aeruginosa*—*Pseudomonas aeruginosa*.

**Table 2:** Summary of six years microbiological water quality.

Microbiological Analysis	Mean	SD	Min	Median	Q3	D9	Max
Total culturable aerobic bacteria at 36 °C (N = 628)	0.8	6.2	0	0	0	1	140
Total culturable aerobic bacteria at 22 °C (N = 628)	0.7	7.3	0	0	0	0	156
*Legionella* sp. (*n* = 626)	0.0	0.0	0	0	0	0	0
*Pseudomonas aeruginosa* (N = 628)	0.0	0.0	0	0	0	0	0
Total coliforms (*n* = 614)	0.0	0.0	0	0	0	0	0

SD—standard deviation; Min—minimum; Q3—3rd quartile; D9—9th decile; Max—maximum.
